# Clinical comparison of unilateral biportal endoscopic technique versus open microdiscectomy for single-level lumbar discectomy: a multicenter, retrospective analysis

**DOI:** 10.1186/s13018-018-0725-1

**Published:** 2018-01-31

**Authors:** Seung-Kook Kim, Sang-Soo Kang, Young-Ho Hong, Seung-Woo Park, Su-Chan Lee

**Affiliations:** 1 The Spine Center, Himchan Hospital, 118 Yongdam-ro, Yunsoo-gu, Incheon, 21927 South Korea; 2 Department of Neurosurgery, College of Medicine, Kangwon National University, Chuncheon, South Korea; 3 Department of Orthopedic Surgery, Leaders Hospital, Seoul, South Korea; 4 Department of Neurosurgery, Bareun-Sesang Hospital, Kyoungki, South Korea; 5 Joint and Arthritis Research, Orthopedic Surgery, Himchan Hospital, Seoul, South Korea

**Keywords:** Arthroscopy, Endoscopic spine surgery, Herniated lumbar disc, MISS, Lumbar disc, Minimally invasive spine surgery, BESS, UBE

## Abstract

**Background:**

The unilateral biportal endoscopic (UBE) technique is a minimally invasive procedure for spinal surgery, while open microscopic discectomy is the most common surgical treatment for ruptured or herniated discs of the lumbar spine. A new endoscopic technique that uses a UBE approach has been applied to conventional arthroscopic systems for the treatment of spinal disease. In this study, we aimed to compare and evaluate the perioperative parameters and clinical outcomes, including recovery from surgery, pain and life quality modification, patient’s satisfaction, and complications, between UBE and open lumbar microdiscectomy (OLM) for single-level discectomy procedures.

**Methods:**

This study included 141 patients with degenerative disc disease requiring discectomy at a single level from L2–L3 to L5–S1. A total of 60 and 81 patients underwent UBE and OLM, respectively. Analysis was based on comparison of perioperative metrics, operation time (OT); estimated blood loss (EBL); length of hospital stay (HS); clinical outcomes, including assessment using the Visual Analogue Scale (VAS) and Oswestry Disability Index (ODI); patient satisfaction (the MacNab score); and the incidence of reoperation and complications.

**Results:**

The study cohort was 56.7% women, and the mean patient age was 50.98 ± 18.23 years. The mean VAS (the back and leg), MacNab score, and ODI improved significantly from the preoperative period to the last follow-up (12.92 ± 3.92) in both groups (*p* < 0.001). One week after operation, the back VAS score in the UBE group showed significantly more improvement than that in the OLM group. However, the 1-week, 3-month, and 12-month VAS (the back and leg), ODI improvement, modified MacNab score, and OT were not significantly different between the two groups. In the UBE group, EBL (34.67 ± 16.92) was smaller and HS (2.77 ± 1.2) was shorter than that of the OLM group (140.05 ± 57.8, 6.37 ± 1.39). However, OT (70.15 ± 22.0) was longer in the UBE group than in the OLM group (60.38 ± 15.5), and the difference was statistically significant. Meanwhile, the differences in the rate of surgical conversion and complications between the two groups were not statistically significant.

**Conclusions:**

The UBE for single-level discectomy yielded similar clinical outcomes to OLM, including pain control, functional disability, and patient satisfaction, but incurred minimal EBL, HS, and postoperative back pain.

**Trial registration:**

Not applicable.

**Electronic supplementary material:**

The online version of this article (10.1186/s13018-018-0725-1) contains supplementary material, which is available to authorized users.

## Background

Lumbar disc herniation (LDH) is a clinically symptomatic condition caused by the compression of the spinal nerve root from a protruded disc material. Almost 70–85% of patients experience at least one episode of lower back pain with or without leg pain during their lives [1]. Some studies have reported that LDH can be naturally absorbed [2, 3]. However, surgery is required when symptoms refractory to medical treatment or combined neurological deficits, including sensory or motor problem, persist. The current standard surgery for LDH is open lumbar microdiscectomy (OLM) with partial laminotomy. However, OLM results in increased risks of postoperative spinal instability and chronic back pain [4]. This procedure is more invasive and is similar to open discectomy. OLM requires bone removal, entrance to the spinal canal, manipulation of neural and vascular tissues, and large fenestration to the annulus. Currently, the popularity of minimally invasive spine surgery for the treatment of LDH is growing. Percutaneous endoscopic discectomy is a minimally invasive spinal surgery (MISS) technique that has several advantages over OLM, including preservation of bony and muscular structure, shorter hospital stay (HS), and a smaller incision [5–7]. A new endoscopic technique that uses a unilateral biportal endoscopic (UBE) approach has been applied to conventional arthroscopic systems for spinal disease [8, 9]. The arthroscopic discectomy technique, as described by Kambin [10], is different from the other MISS procedures because it allows for extraction of the offending herniated fragments from the posterior intervertebral disc. Patient satisfaction was rated at 87% [11, 12], and the radiologic success rate was 16 out of 18 case series [11–13]. However, these reports were published before 2000, and a detailed analysis on pain and patient satisfaction over time is yet to be performed. High-definition (HD) endoscopic visualization has been available since 2007, allowing for better illumination and tissue identification compared with previous standard definition visualization [14, 15]. The clinical results of these techniques with respect to comparisons of various parameters have not been analyzed to date. To the best of our knowledge, this is the first report on the evaluation of the clinical results of these techniques since they were introduced.

## Methods

This study aims to compare the differences in the 1-year postoperative clinical course in terms of perioperative parameters, such as pain control, quality of life modification, and patient satisfaction between biportal endoscopic and traditional microscopic techniques. This is a case control study conducted at Himchan Hospital, Incheon, Korea; Leaders Hospital, Seoul, Korea; and Bareun-Sesang Hospital, Kyoungki, Korea. We enrolled 141 patients who underwent surgery for the treatment of LDH between May 2016 and October 2016; 60 consecutive patients were treated with UBE by three surgeons (Dr. S. Kim, Dr. S. Kang, and Dr. Y. Hong), while 81 consecutive patients were treated with OLM by two surgeons (Dr. S. Kim and Dr. Y. Hong). The inclusion criteria were (1) back or radiating pain related to LDH, (2) symptom persistence of more than 4 weeks, and (3) magnetic resonance (MR) images correlated to the symptoms. The exclusion criteria were as follows: (1) foraminal or extraforaminal disc involvement, (2) recurred LDH, (3) motion instability (defined as > 3 mm translation or > 5° angulation), (4) spondylolisthesis more than Meyerding grade II, (5) cauda equine syndrome, and (6) comorbid tumorous or infectious conditions. All participating institutions received approval from their respective institutional review board (KNU07-1112), and all patients provided written informed consent. The data were collected starting from the preoperative period until 12 months postoperative. Pain intensity, patient satisfaction, and quality of life as analyzed using the Visual Analogue Scale (VAS), modified MacNab score, and Oswestry Disability Index (ODI), respectively, were investigated at 1-week, 3-month, and 12-month postoperative follow-ups.

Clinical outcomes were evaluated using the back and leg VAS (0–10) and the ODI (0–100%). Patient satisfaction was assessed via modified MacNab criteria (excellent, good, fair, and poor). Perioperative data (length of operation time (OT) and HS, estimated blood loss (EBL), and complications) were assessed via video records of the endoscopic and microscopic operation and clinical charts. Radiologic outcomes were evaluated using the pre- and 3-day postoperative MR images.

### Surgical techniques

#### Unilateral UBE discectomy

The UBE was performed under epidural anesthesia with the patient in the prone position on a C-arm fluoro-radiolucent table. Conscious sedation with sedative analgesia and music listening was allowed, which enabled the surgeon to avoid injuring the neural structures.

During the procedure, we used 0° or 30° 4-mm rigid arthroscope (Hopkins® arthoroscope Storz, El Segundo, USA), 3.5-mm spherical burr (Dyonics® drill, Smith & Nephew, Andover, USA; Smith & Nephew, London, UK), 3.5-mm radiofrequency (RF) ablation probe (RF® Ablation system, Stryker, Kalamazoo, MI, USA), a pressure pump irrigation system (Smith & Nephew), and standard instruments for open laminectomy, such as hook dissectors, Kerrison punches, Rotating Kerrison punches (Osteo Rongeur, Koros, USA, CA, USA), and pituitary forceps.

The surgery proceeded as follows (see Additional file 1).Additional file 1: Supplementary video clip.The supplementary video clip demonstrates the full process for the endoscopic unilateral biportal endoscopic (UBE) technique. (1) We performed unilateral partial laminotomy with automated drill. (2) Using a small laminotomy window, the interlamina was dissected with a radiofrequency probe. (3) Partial removal of the yellow ligament was performed with Kerrison punches. (4) Adhesion removal and disc dissection were done with hook dissector. (5) Ruptured disc removal and (6) disc space exploration and confirming the nerve root exposure were performed using a pituitary forcep and hook dissector (MP4 104919 kb).

First, the two portal skin entry points were confirmed using preoperative axial MR images or plain anteroposterior (AP) radiographs to determine the optimal operation route. Then, the target disc was identified under the discographic images. In the left side approach, the insertion point for the endoscope (endoscopic portal) was 1–1.5 cm lateral to the midline in the lower margin of the upper lamina, while the upper margin of the lower lamina was the insertion point for surgical instruments (instrumental portal). The endoscopic portal was used for continuous irrigation and for viewing of the surgical procedure, while the instrumental portal was used for instrument manipulation and removal of the ruptured disc. After a serial dilator was inserted through the caudal portal, the muscle was dissected with an RF probe through the instrumental portal. The lower lamina of the upper lumbar spine and upper lamina of the lower lumbar spine were partially removed via an automated drill and Kerrison punches (partial laminotomy). The interlaminar ligament was then dissected using an RF probe and removed using rotating Kerrison punches. Annulotomy, disc fragment dissection, and ruptured fragment removal were performed using pituitary forceps and Kerrison punches. Decompressed root confirmation and disc space exploration were performed using a 90^°^ hook dissector. The muscle and skin were sutured using a 2:0 absorbable suture (Vycryl®) and reinforced skin closure (Steri-Strip®, 3M, Inc.), Maplewood, MN, USA).

#### OLM

OLM was performed under general or spinal anesthesia. The surgical procedure followed the standard method using a tube or Caspar retractor system [16, 17]. The procedure was performed with the patient in a prone position on a radiolucent table. The incision point was at the inferior edge of the superior lamina of the lesion side in the AP view and parallel to the disc space in the lateral view. After creating a 3-cm incision in the midline, the fascia was dissected to the lateral edge of the inferior articular facet. Soft tissues, including the paraspinal muscles, were cleaned using a monopolar cautery system (Bovie® Medical, Inc., Purchase, NY, USA) to expose the ligamentum flavum. After partial laminotomy of the lower lamina of the upper lumbar spine and upper lamina of the lower lumbar spine, the ligamentum flavum was removed for disc discrimination. Then, the instruments were advanced to the epidural space and the dura margin, and the nerve roots were exposed. The root was retracted, and epidural dissection was performed. The protruded disc particles were found and removed with pituitary forceps and Kerrison punches. The mobility of the root was checked using a hook dissector after the pathologic disc particles were removed. Wound closure was performed using 1:0, 2:0, and 4:0 absorbable sutures (Vycryl®) and a skin stapler.

### Statistical analysis

Statistical analyses were performed using SPSS for Windows (version 22.0; SPSS, Inc., Chicago, IL, USA). An independent sample *t* test (two sided) and Mann-Whitney test were used to compare numerical data between groups, such as VAS, ODI, OT, EBL, HS, postoperative complication, and follow-up duration. Fisher exact test and *χ*^2^ test were used to compare categorical variables including sex, disc location, operation level, modified MacNab score, motor weakness, complication, and surgery conversion between groups. Changes in periodical variables from the preoperative period to each postoperative time were measured using Wilcoxon signed rank test and paired sample *t* test. A *P* value less than 0.05 was considered statistically significant.

## Results

**Fig. 1:**
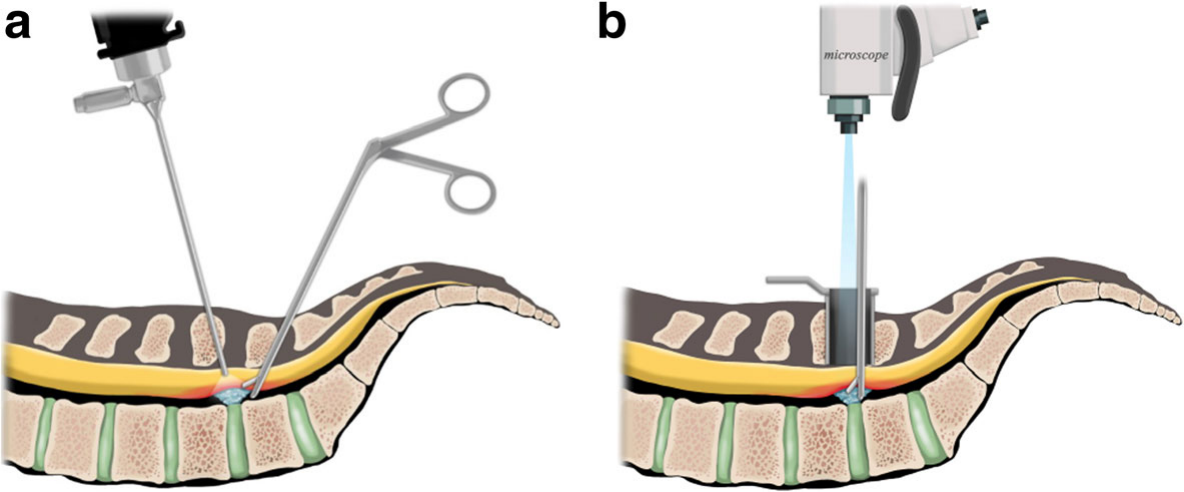
Illustration of **a** unilateral biportal endoscopic discectomy and **b** open microscopic discectomy

A total of 146 patients who underwent spinal surgery were enrolled in the present study. Two of the 62 patients who underwent UBE and three of the 84 who underwent OLM were excluded because they were lost to follow-up. Consequently, we enrolled 60 and 81 patients who underwent UBE and OLM, respectively. The patients’ demographic and preoperative characteristics (Table 1) were not statistically different. The schematic differences between the two procedures are depicted in Fig. 1a, b.

**Table 1:** Patients’ demographic data

		UBE (*n* = 60)	OLM (*n* = 81)	*p* value
Age (years)		46.60 ± 14.18	54.22 ± 20.21	0.121
Sex (%)	M	37 (61.7)	24 (29.6)	0.072
	F	23 (38.3)	57 (70.4)	
Symptom (%)	Pain only	18 (30.0)	25 (30.9)	0.531
	Pain and weakness	42 (70.0)	56 (69.1)	
Symptom duration (weeks)		4.67 ± 0.72	4.60 ± 0.71	0.591
Follow-up duration (months)		12.60 ± 1.03	12.84 ± 1.30	0.225
Disc location (%)	Central	11 (18.3)	18 (22.2)	0.365
	Paracentral	49 (81.7)	63 (77.8)	
Disc level (%)	L2–3	1 (1.7)	4 (4.9)	0.444
	L3–4	2 (3.3)	4 (4.9)	
	L4–5	34 (56.7)	36 (37.0)	
	L5-S1	23 (38.3)	37 (45.7)	

*UBE* unilateral biportal endoscopy, *OLM* open lumbar microscopy

**Fig. 2:**
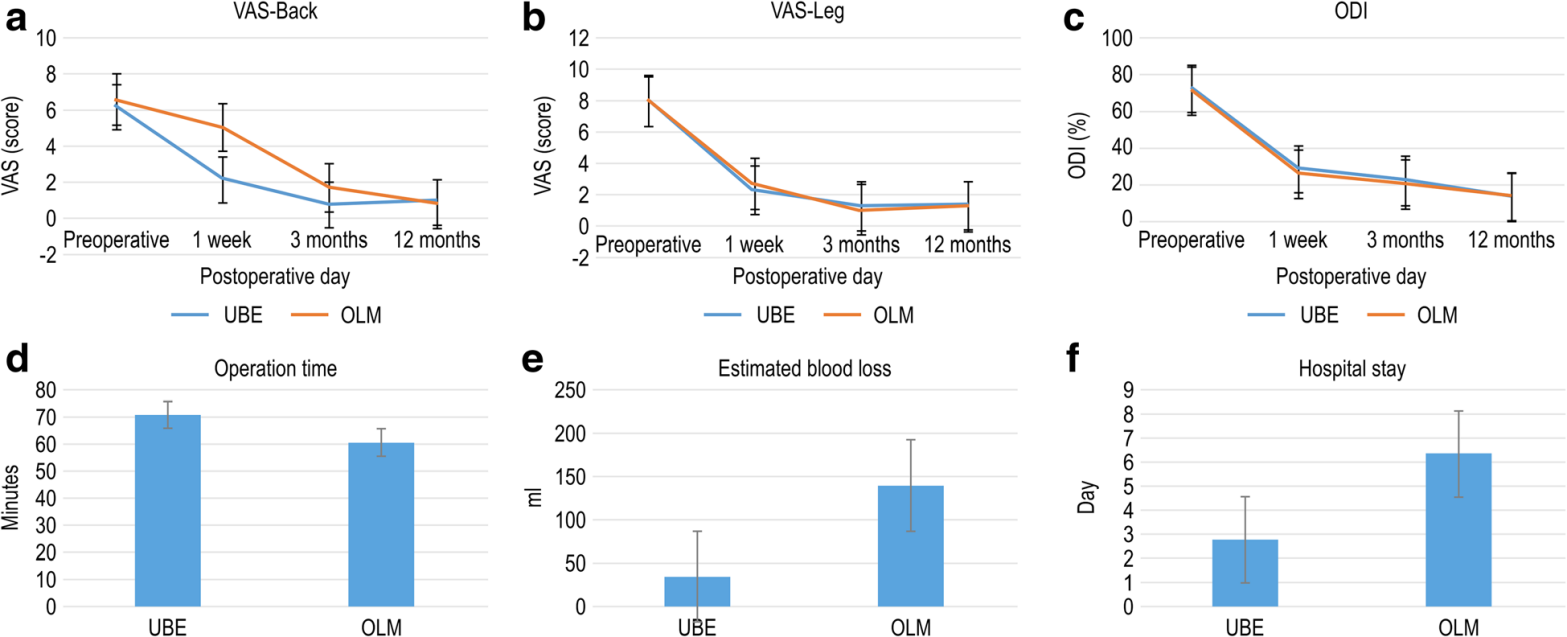
Clinical outcomes during follow-up (1 week, 3 months, and 12 months) and perioperative data. Visual Analogue Scale (VAS) scores for back pain (**a**), VAS for leg pain (**b**), Oswestry Disability Index (ODI, %) (**c**), operation time (minutes) (**d**), estimated blood loss (ml) (**e**), hospital stay (days) (**f**)

**Fig. 3:**
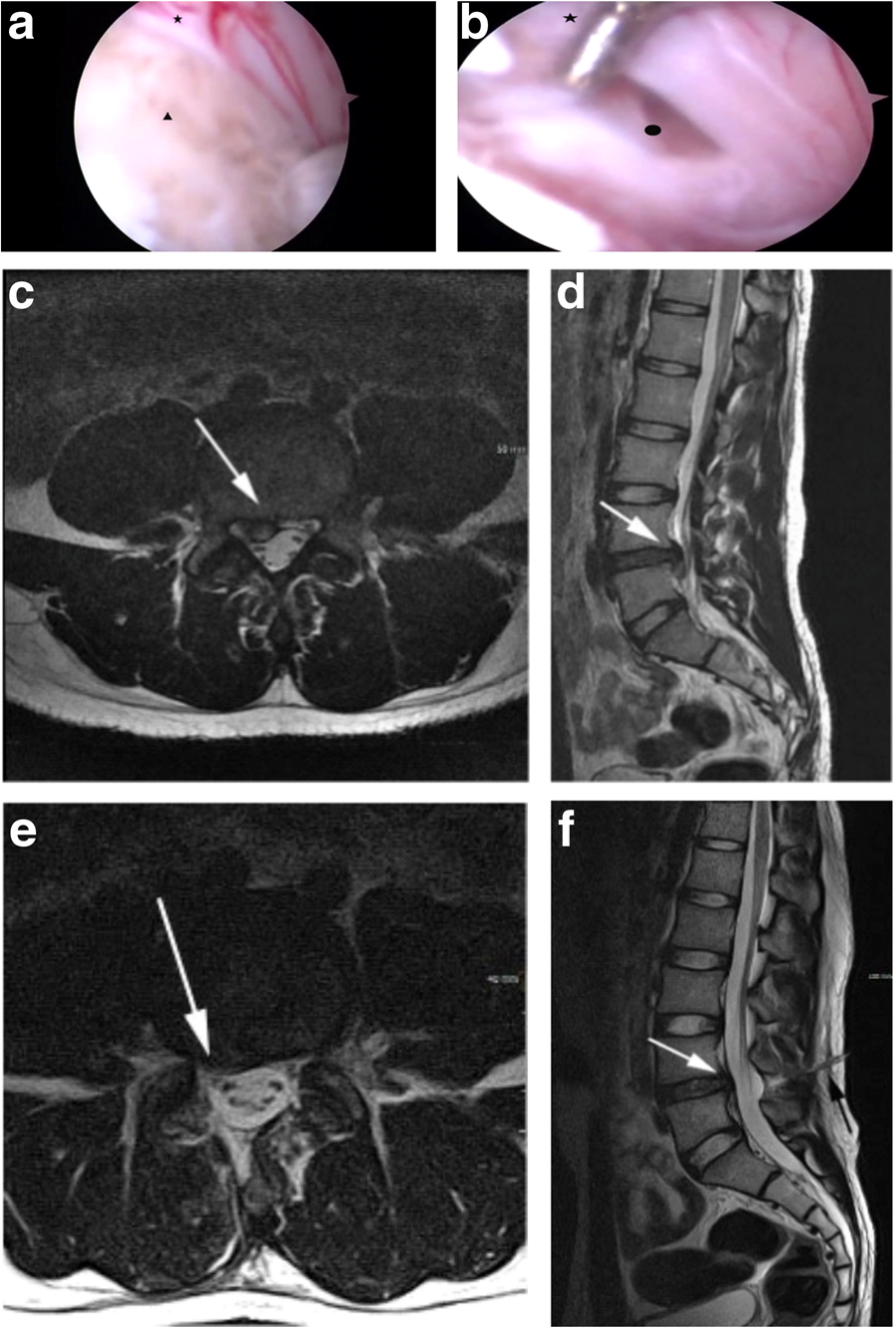
Large disc herniation at the L4–5 level. Intraoperative imaging during unilateral biportal endoscopic (UBE) discectomy of a 28-year-old man presenting with severe back and right leg radiating pain. The herniated disc triangle compressing the thecal sac and traversing nerve root star (**a**), thecal sac, traversing nerve root star, and the posterior ligament circle were freely movable after disc fragment removal (**b**). Magnetic resonance (MR) axial image shows paracentral disc herniation and compression in the thecal sac and L5 traversing nerve root (white arrow) (**c**). Sagittal MR image shows down migrated disc compressing the thecal sac (white arrow) (**d**). After UBE discectomy, postoperative axial MR image shows decompressed thecal sac and traversing L5 nerve root (white arrow) (**e**). Postoperative sagittal MR imaging shows removed herniated disc (white arrow) and minimally invasive instrumental pathway for discectomy (black arrow) (**f**)

The clinical outcomes and operative findings are shown in Table 2. In both groups, postoperative back and leg pain and ODI were significantly improved (*p* < 0.001, Fig. 2a–c). Improvements in back pain 1 week after operation were significantly different between the UBE and OLM groups (4.05 ± 1.6 vs. 1.25 ± 1.7, *p* < 0.001). The mean OT was significantly longer in the UBE group (70.15 ± 22.0 min, *p* = 0.002) than in the OLM group (60.38 ± 15.5 min, Fig. 2d). The mean blood loss in the UBE group was significantly less than in the OLM group (34.67 ± 16.9 ml vs. 140 ± 57.8 ml, *p* < 0.001, Fig. 2e). The mean HS was significantly shorter in the UBE group than in the OLM group (2.77 ± 1.2 d vs. 6.37 ± 1.4 d, *p* = 0.005, Fig. 2f). After the ruptured or protruded disc was dissected, the compressing materials were removed (Fig. 3a). A decompressed traversing root and thecal sac indicated completion of operation (Fig. 3b). Compared with preoperative MRI (Fig. 3c), postoperative (Fig. 3d) MRI indicated relieved pathologic condition (Fig. 3e) with limited muscle injury radiologically (Fig. 3f). The ruptured disc fragment was completely removed in all cases except in three cases of UBE that required conversion to OLM. The surgery was modified due to blurred field of view from the bone and epidural bleeding. Controlling bleeding in the microscopic view is important because the RF probe and bone can be difficult to manipulate due to vision disturbance. No serious complications, including cauda equine syndrome, were observed. Two cases of cerebrospinal fluid leakage occurred, which were treated with conservative treatment including bed rest and fluid replacement. Only one case of operative site infection occurred in the OLM group, which was controlled using 3rd-generation antibiotics, such as cefotaxime.

**Table 2:** Comparison of clinical outcomes of UBE and OLM for LDH

	UBE	OLM	*p* value
Pre-op VAS back	6.22 ± 1.5	6.33 ± 1.5	0.263
Pre-op VAS leg	7.93 ± 1.0	7.98 ± 1.0	0.808
Post-op VAS back	0.93 ± 0.7	0.85 ± 0.7	0.657
Post-op VAS leg	1.28 ± 1.0	1.27 ± 1.0	0.945
Pre-op ODI	70.15 ± 1.0	71.85 ± 8.4	0.815
Post-op ODI	14.5 ± 11.9	13.95 ± 11.5	0.549
Improvement of VAS back (1 week)	4.05 ± 1.6	1.25 ± 1.7	0.001*
Improvement of VAS back (12 months)	5.28 ± 1.80	5.28 ± 1.80	0.504
Improvement of VAS leg (1 week)	5.86 ± 1.6	5.60 ± 1.5	0.326
Improvement of VAS leg (12 months)	6.65 ± 1.5	6.70 ± 1.4	0.914
Improvement of ODI back (1 week)	45.67 ± 12.3	45.18 ± 12.8	0.824
Improvement of ODI (12 months)	57.90 ± 13.5	58.17 ± 15.6	0.782
Modified MacNab score (%)	75.35 ± 0.5	68.46 ± 0.5	0.082
OT	70.15 ± 22.0	60.38 ± 15.5	0.002*
EBL	34.67 ± 16.9	140 ± 57.8	0.001*
HS	2.77 ± 1.2	6.37 ± 1.4	0.005*
Complications (%)	3 (3.7)	2 (3.3)	0.640

*UBE* unilateral biportal endoscopy, *OLM* open lumbar microscopy, *LDH* lumbar disc herniation, *VAS* Visual Analogue Scale (0–10), *ODI* Oswestry Disability Index (0–100%), *improvement*, the difference between preoperative and postoperative results, modified MacNab (1, excellent; 2, good; 3, fair; 4, poor), *OT* operation time, *EBL*, estimated blood loss, *HS* hospital stay (days)

*p < 0.05

## Discussion

As a form of MISS, UBE demonstrated several advantages and one disadvantage in the present study. First, it showed superiority in terms of short-term back pain recovery, a small volume of intraoperative blood loss, and less HS. Second, improvements in short-term leg pain and long-term back and leg pain, modification of the quality of life (ODI), patient satisfaction (modified MacNab score), and complication rate were similar to that of OLM. However, OT was longer in UBE than in OLM, but this is its only disadvantage in the present study. These results indicate that UBE can be used to minimize tissue damage, although several limitations, such as controlling bleeding, need to be overcome.

Although conventional open laminotomy and discectomy is an effective way for symptomatic herniation, muscle and ligament injury from surgery can lead to postoperative back pain and muscle atrophy [18]. Therefore, more time may be required for functional recovery and pain control after OLM. Postoperative back pain following mechanical trauma due to OLM has already been reported. Dvorak et al. reported that 70% of patients experienced back pain after conventional discectomy during long-term follow-up [19]. Parker et al. also reported that 32% of patients suffered back pain after conventional discectomy, and 9% of cases underwent fusion surgery for pain control [20]. Vodicar et al. reported that invasive procedures, including endplate perforation, decrease vertebral height and worsen back pain in the postoperative period [21]. Scarring of the epidural space can be problematic [22–24]. It may become clinically symptomatic and make revision surgery more difficult because of the connection of the thecal sac to the paravertebral muscle structures [25, 26]. As such, MISS techniques, such as transforaminal and interlaminar approach percutaneous endoscopic lumbar discectomy (PELD), have been developed to minimize injury to the posterior musculo-ligamentous structures [27, 28].

Uniportal transforaminal and interlaminar PELD are both good surgical methods. They can protect the posterior structures, such as the upper and lower laminas, ligamentous structure, and muscles, better than OLM. Although these procedures can remove soft disc herniation and ruptured LDH without foraminal obstruction, they have limited indications due to the restricted movements of the instruments and obstructed intervertebral foramen following degenerative changes. Microendoscopic discectomy is regarded as an alternative to OLM because it produces few traumas to soft tissues and results in rapid recovery and less intraoperative blood loss [29]. However, this technique requires the same exposure of muscle and bone and basic skills with that of conventional OLM, such as the use of a dilator and tubular retractor [29]. By contrast, UBE can achieve high-resolution visualization at only a small muscle dissection and use almost all laminectomy instruments without restriction. HD endoscopic vision makes disc dissection easier, and ruptured fragment removal and manipulation is possible as in the conventional technique. Because the same instruments are used while allowing for a more detailed view than in microscopic surgery, favorable radiologic outcomes can be achieved. UBE is a new method that combines the advantages of interlaminar endoscopy and microscopic surgery. The use of the uniportal system is limited because of the combined channel (viewing and instrumental) that limits the independent movement of instruments. By contrast, the UBE system uses independent channels for instruments; thus, movements are not restricted. Furthermore, instruments for both 0^°^ or 30^°^ arthroscopy for the knees and shoulders and standard laminectomy are used and additional devices are no longer needed. Moreover, the endoscopic trajectory is the same as that in conventional operation; thus, an experienced microscopic spine surgeon can achieve the necessary surgical skills without a steep learning curve [30].

Kambin et al. reported a high rate of 87% patient satisfaction for arthroscopic disc surgery [12]. The rating was based on pain reduction, medication changes, and lifestyle modifications. However, this study did not use universally accepted assessment scales such as VAS, ODI, and modified MacNab score. Casey et al. assessed the radiologic outcomes of arthroscopic discectomy and found that the success rates based on CT and MRI were 88.9% (*n* = 18) and 85.7% (*n* = 12), respectively [13]. However, this study did not perform a control analysis, and only radiologic outcomes were assessed. A recent study by Um et al. reported the outcomes of UBE after development of HD endoscopic vision [8]. The study showed that the ODI score decreased from 67.2 ± 1.7 to 24.3 ± 8.5, and the VAS for leg pain decreased from 8.3 ± 1.1 to 2.4 ± 1.1. This study showed favorable outcomes from UBE, which are consistent with our study. However, control group analysis was not performed, and the operation detail was not discussed. The present study is characterized by a detailed evaluation of the operation, analysis of controls, and evaluation according to the perioperative period. We also described the drawback of this surgery, which was prolonged OT.

Technical advances in the surgical techniques of LDH now permit a fully endoscopic procedure under continuous irrigation. This can provide optimal advantages for a MISS procedure [14] that became possible with more tissue-sparing techniques, which are being applied increasingly [31]. Compared with conventional OLM, UBE has the advantage of less intraoperative blood loss and postoperative back pain and relatively shorter HS due to the preservation of the back muscle and a smaller incision. These advantages extend the scope of lumbar spinal stenosis [30], degenerative diseases of the cervical spine, and even short-level fusion surgeries [8]. Through high-resolution video equipment, preserving the facet joint and ligament complex and lessening nerve traction is now possible. Another advantage is that UBE preserves the epidural vessel and discal tissues, avoiding annular incision with the knife. The combination of these advantages results in improved quality of life (ODI score). In terms of patient satisfaction, the modified MacNab score in UBE was equivalent to that in conventional OLM despite prolonged OT. This result may be due to the tissue-sparing nature of the procedure, rapid pain recovery, short HS, favorable pain outcomes, and improved quality of life.

Our results show that OT is longer in UBE than OLM primarily because most surgeons have been used to microscopic procedures for a long time. In UBE, only the right hand is in the instrument portal because the working portal is used only for the instruments; thus, the surgeon cannot use both hands, making it difficult to control bleeding and prolonging the OT. However, more surgical experience will reduce the OT.

The limitations of this study are its retrospective nature, small sample size, and short follow-up period. In addition, because of the nature of retrospective studies, selection bias seems to be intrinsic by patients’ preferences and the surgeon’s experience may be influenced the outcomes. However, the results show that UBE can be an alternative surgery to OLM based on the favorable clinical results and the convenience from the new endoscopic instruments. Adequate randomized prospective studies for UBE are required to verify the present results.

## Conclusions

UBE can be an effective treatment modality for LDH. The anatomic trajectory and endoscopic view is similar to that of conventional discectomy. UBE for single-level discectomy has several advantages; the similar sufficient and direct fragmentectomy and discectomy to that in open microdiscectomy resulted in the same clinical outcomes, including pain improvement, functional disability, and patient satisfaction and minimal EBL, HS, and postoperative back pain, while preserving the spinal tissues. Considering the bleeding tendency and adequate indications, UBE is a highly feasible alternative to conventional microscopic operation.

## Abbreviations

AP: Anteroposterior
EBL: Estimated blood loss
HD: High definition
HS: Hospital stay
LDH: Lumbar disc herniation
MISS: Minimally invasive spinal surgery
MR: Magnetic resonance
ODI: Oswestry Disability Index
OLM: Open lumbar microdiscectomy
OT: Operation time
PELD: Percutaneous endoscopic lumbar discectomy
RF: Radiofrequency
UBE: Unilateral biportal endoscopy
VAS: Visual Analogue Scale
